# Enhanced Performance and Diffusion Robustness of Phase-Change Metasurfaces via a Hybrid Dielectric/Plasmonic Approach

**DOI:** 10.3390/nano11020525

**Published:** 2021-02-18

**Authors:** Joe Shields, Carlota Ruiz de Galarreta, Jacopo Bertolotti, C. David Wright

**Affiliations:** College of Engineering Mathematics and Physical Sciences, University of Exeter, Exeter EX4 4QF, UK; js1116@exeter.ac.uk (J.S.); cr408@exeter.ac.uk (C.R.d.G.); j.bertolotti@exeter.ac.uk (J.B.)

**Keywords:** active metasurfaces, phase-change metasurfaces, hybrid dielectric/plasmonic metasurfaces, gold diffusion in phase-change materials

## Abstract

Materials of which the refractive indices can be thermally tuned or switched, such as in chalcogenide phase-change alloys, offer a promising path towards the development of active optical metasurfaces for the control of the amplitude, phase, and polarization of light. However, for phase-change metasurfaces to be able to provide viable technology for active light control, in situ electrical switching via resistive heaters integral to or embedded in the metasurface itself is highly desirable. In this context, good electrical conductors (metals) with high melting points (i.e., significantly above the melting point of commonly used phase-change alloys) are required. In addition, such metals should ideally have low plasmonic losses, so as to not degrade metasurface optical performance. This essentially limits the choice to a few noble metals, namely, gold and silver, but these tend to diffuse quite readily into phase-change materials (particularly the archetypal Ge_2_Sb_2_Te_5_ alloy used here), and into dielectric resonators such as Si or Ge. In this work, we introduce a novel hybrid dielectric/plasmonic metasurface architecture, where we incorporated a thin Ge_2_Sb_2_Te_5_ layer into the body of a cubic silicon nanoresonator lying on metallic planes that simultaneously acted as high-efficiency reflectors and resistive heaters. Through systematic studies based on changing the configuration of the bottom metal plane between high-melting-point diffusive and low-melting-point nondiffusive metals (Au and Al, respectively), we explicitly show how thermally activated diffusion can catastrophically and irreversibly degrade the optical performance of chalcogenide phase-change metasurface devices, and how such degradation can be successfully overcome at the design stage via the incorporation of ultrathin Si_3_N_4_ barrier layers between the gold plane and the hybrid Si/Ge_2_Sb_2_Te_5_ resonators. Our work clarifies the importance of diffusion of noble metals in thermally tunable metasurfaces and how to overcome it, thus helping phase-change-based metasurface technology move a step closer towards the realization of real-world applications.

## 1. Introduction

The field of metasurfaces has expanded rapidly over the past decade due to the promise of arbitrary control over electromagnetic waves spanning the frequency spectrum in the microwave-to-optical range. Metasurfaces are engineered two-dimentional surfaces consisting of subwavelength resonant building blocks (often termed as “meta-atoms”), made of plasmonic (metallic) and/or dielectric materials that are periodically or randomly arranged [1,2]. Since the far field of a beam that interacts with a surface is defined by electric and magnetic components that are tangential to that surface (near field), each discrete area (i.e., each meta-atom) effectively behaves as a secondary field source. Following the Huygens principle, the resulting far-field characteristics can be controlled by both the relative position (spacing) of the secondary sources and engineering their local electromagnetic near-field interactions [1]. As a result, a metasurface is specifically designed to provide precise local or global subwavelength amplitude, phase, and polarization control of light by the judicious selection of the shape, dimensions, spacing, and constituent materials that comprise the meta-atoms, which allows for them to mimic and even outperform classical optical components [1,3]. However, although optical metasurfaces emerged as a flexible design platform that offered clear advantages over classical optical components, their optical performance is fixed by design, and thus locked in at the fabrication stage, that is, their electromagnetic properties are static, so a particular metasurface has a repeatable effect on optical beams [4]. To overcome such a limitation, a range of approaches to realize reconfigurable and dynamically tunable (or active) metasurfaces were proposed by the scientific community over the past decade [4,5]. These include optically, electrically, thermally, chemically, and mechanically tunable metasurfaces, among which thermal tuning—employing materials of which the optical properties are thermally sensitive (such as silicon, vanadium dioxide, or chalcogenide phase-change materials)—is perhaps the preferred approach to date [4,6,7]. In particular, thermal tuning employing chalcogenide phase-change materials (PCMs), such as the archetypal compound Ge_2_Sb_2_Te_5_ (GST), is one of the most promising techniques to yield dynamically tuneable metasurfaces [4]. Chalcogenide PCMs are a group of materials of which the refractive index can be controlled by causing them to transition from an amorphous to a crystalline state by using a heat stimulus. While crystallization requires moderately elevated temperatures (around 180 to 400 °C for GST, depending on heating rate [8]), reamorphization requires the PCM to be heated above its melting temperature (630 °C for GST [8]), followed by a quick cooling rate to retain the less energetically favorable amorphous phase. In the case of traditional PCMs such as GST, reamorphization can be achieved via the use of short electrical or optical pulses (e.g., tens to hundreds of nanoseconds in PCM-based memories) [4,8] over small PCM volumes surrounded by good thermal conductors to avoid thermal insulation (and thus slow cooling rates) [9,10]. Access to intermediate or fractionally crystallized PCM states can also be achieved by appropriate excitation (heat stimulus), and can allow for both increased degrees of freedom (multilevel states) and more precise control over the PCM’s optical properties [9,11,12,13].

**Figure 1:**
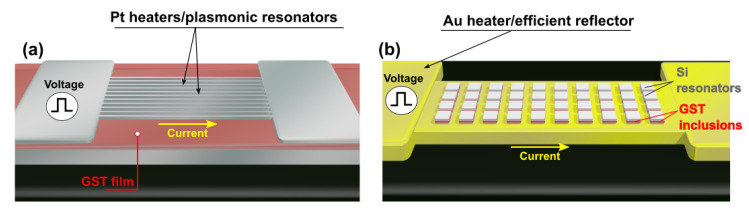
(**a**) Schematics of electrical switching of reconfigurable phase-change plasmonic metasurfaces where Pt resonators are simultaneously used as resistive heaters, (as described in [24]). (**b**) Concept of electrical switching of reconfigurable hybrid dielectric/plasmonic phase-change metasurfaces where highly efficient plasmonic metals (here Au) are simultaneously used as bottom heater and reflective plane.

Recently, phase-change metasurfaces based on metallic architectures that exploit plasmonic resonators for local and global amplitude and/or phase control were proposed [14,15,16,17,18,19], some of them with in situ (electrical) switching potential via the use of metallic elements, such as resistive heaters [16,17,18,19]. However, such structures suffered from fundamental plasmonic losses, which resulted in reductions in both efficiency and attainable resonant Q factors, thus reducing device functionality. In such devices, PCMs were used as a tunable “insulator” medium, whereas metallic layers can be simultaneously used to excite gap plasmon resonances, and as a resistive heater to switch the PCM layer between amorphous and crystalline states [17]. In this context, metals such as Au, Ag, and Al are the prefered options for the realization of metal-insulator-metal (MIM) metasurfaces mainly due to their excellent plasmonic properties in the near-infrared-to-THz range. However, the melting point of Al (660 °C) is only marginally above the melting point of traditional Ge–Sb–Te alloys; thus, the long-term degradation or deformation of Al is likely if used as a heating element. On the other hand, Au and Ag possess higher melting points and seem better a priori options for the realization of robust plasmonic heaters. However, Au tends to readily diffuse into PCMs [20] and other commonly used metasurface materials, such as silicon [21,22] (with the potential for the formation of gold tellurides in the former [20] and gold silicides in the latter [23]) at elevated temperatures. This can lead to a significant degradation of optical properties and performance in device applications [20]. As depicted in Figure 1a, alternative higher-melting-point metals that exhibit lower thermally activated diffusion (such as Pt, TiN, or W) were also proposed, but unfortunately at the cost of reduced optical performance (lower optical efficiencies) due to the presence of higher plasmonic lossess in such metallic elements [16,17,24].

Another recently proposed active metasurface approach relied on the combination of all-dielectric silicon metasurfaces with deeply subwavelength-sized PCM inclusions, which provided a promising way of manipulating the amplitude and phase of light with high efficiency via the excitation of Mie-like resonances, free from plasmonic losses [9]. However, despite providing superior optical efficiencies, the in situ reversible switching of the phase-change layer was, in this case, complicated by the lack of metal layers to provide heating elements (as shown in Figure 1a). Such a limitation could be overcome by the use of hybrid dielectric/plasmonic nanoantennas based on high-index dielectric resonators lying on metallic planes, which were suggested as a way to provide the best of both (plasmonic and all-dielectric) metasurface worlds [4,25]. This implies that a properly designed metasurface geometry achieves a high-efficiency optical phase and amplitude manipulation offered by purely dielectric metasurfaces [26,27] in tandem with the properties of plasmonic elements: superior electric-field confinement, superabsorption, and the excitation of surface plasmon polaritons [28]. This makes hybrid dielectric/plasmonic nanoantennas a highly versatile design platform for the realization of devices with a widespread range of functionalities.

As summarized in Figure 1b, we propose the use of hybrid dielectric/plasmonic metasurfaces as a suitable route for highly versatile and thermally tunable metasurfaces. For this purpose, we incorporated a thin GST layer within the body of a cubic silicon nanoresonator lying on a metallic plane. The plane simultaneously acted as a high-efficiency reflective element and resistive heater to induce in situ GST phase transitions [27]. As proof of concept, we designed, fabricated, and tested a set of hybrid metasurfaces capable of absorbing and modulating light in the O (∆λ = 1300–1360 nm) and C (∆λ = 1530–1565 nm) telecommunication bands upon crystallization of the GST layer. Importantly, a number of different configurations of the ground metal/heater plane were investigated with the aim of understanding the effect of thermally activated metal diffusion into the dielectric resonators on the optical performance. Our results showed that metasurfaces fabricated on nondiffusive Al/Al_2_O_3_ bottom metal planes did not exhibit any signs of degradation of their optical performance upon switching (crystallization) of the GST layer. In contrast, metasurfaces fabricated directly onto Au metal planes revealed a catastrophic degradation of optical performance upon GST crystallization, which we believe was due to thermally activated diffusion of Au. However, we showed that the negative impact on optical performance was successfully overcome by adding an ultrathin layer (8 nm) of Si_3_N_4_. This paves the way for the use of high-melting-point plasmonic metals such as Au in the dual role of plasmon generation and the provision of a resistive heating element capable of in situ electrical switching of phase-change metasurfaces. Our findings clarified the importance of considering metal diffusion in thermally tunable metasurfaces, and how to overcome it in the design stage. 

## 2. Materials and Methods

### 2.1. Hybrid Metasurface Design Philosophy

**Figure 2:**
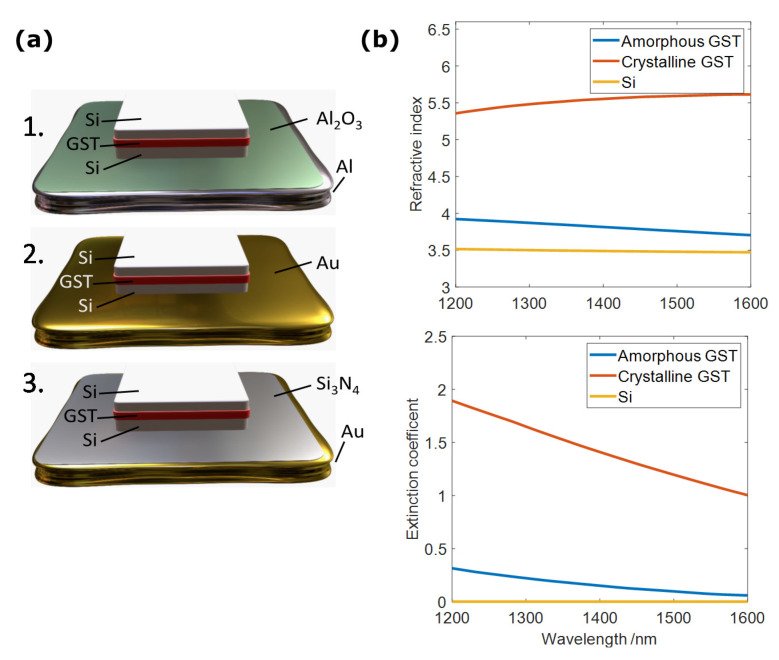
(**a**) Schematics of 3 unit cells (meta-atoms) explored in this work showing Si–GST–Si cubic resonators and different considered bottom planes. (**top**) Al with native Al_2_O_3_ layer, (**middle**) Au, and (**bottom**) Au with an added Si_3_N_4_ layer. These unit cells were repeated in a square lattice arrangement. (**b**) Refractive index and extinction coefficient of GST in amorphous and crystalline phases (measured via ellipsometry), and of silicon (taken from [33]) across spectral range of interest.

Figure 2a shows the unit cells of the three hybrid dielectric/plasmonic designs considered in this work. All consisted of a cubic silicon resonator lying on various metal planes and embedded with a thin layer of GST. As outlined above, the metal planes were intended to play the dual role of plasmon generation and in situ resistive heating to induce the crystallization and reamorphization of the GST layer [27,29]. Instead of using resonators fully made of GST [11], silicon was employed as a way to provide sufficient material volume to excite dielectric and plasmonic resonances [25] while minimizing the amount of GST volume required for a successful reamorphization process [9,18,27]. 

Design 1 (Figure 2a, top) consisted of an aluminum (Al) bottom plane with its characteristic Al_2_O_3_ native oxide layer (4 nm). Aluminum is a cheap, low-loss plasmonic metal that is also CMOS compatible, and, contrary to other low-loss plasmonic metals, it does not suffer from severe diffusion into silicon [30] or PCMs such as GST [18].

For Design 2 (Figure 2a, middle), we considered an Au bottom plane in direct contact with the Si–GST–Si cubic resonator in order to experimentally investigate any negative impact on the optical response of the metasurface due to Au diffusion into the unit cell. Despite Au being an excellent plasmonic material with a higher melting point compared to that of aluminum, its diffusion into phase-change materials severely degraded the optical performance of blanket PCM films [20] and Si/GST systems, as we show later.

Lastly, in Design 3 (Figure 2a, bottom) we incorporated a Si_3_N_4_ layer placed between the Au plane and the Si–GST–Si cubes in order to investigate its potential as a diffusion-preventive barrier. Si_3_N_4_ was chosen due to its excellent thermal stability [31] and good adhesion to Au when deposited via magnetron sputtering. For the phase-change layer, GST was selected due to its large difference in refractive index upon crystallization (∆*n* ∼1.7 and ∆*k* ∼0.9 in the near-infrared). Amorphous GST’s refractive index also closely matches Si at the wavelength range of interest, as shown in Figure 2b. This means that switching GST from its amorphous to crystalline state effectively switches the unit cell from all-Si resonators to Si–GSTc–Si resonators [32]. 

### 2.2. Hybrid-Metasurface Optimization and Analysis

Our hybrid dielectric/plasmonic metasurface devices were designed and modelled with the aid of commercial finite-element analysis package COMSOL Multiphysics^®^ (COMSOL Inc., Stockholm, Sweden). Simulations were carried out in the frequency domain, with light at normal incidence and with transverse magnetic polarization. Floquet periodic boundary conditions were used, and meshing resolution was varied on the basis of the thickness of the used layers and materials. Perfectly matched layers were used at the top and bottom of the simulation space to avoid unwanted reflections from truncations, which thus simulated free space. The optical properties of Au, Al, Al_2_O_3_ and Si_3_N_4,_ were used from [34,35,36,37].

**Figure 3:**
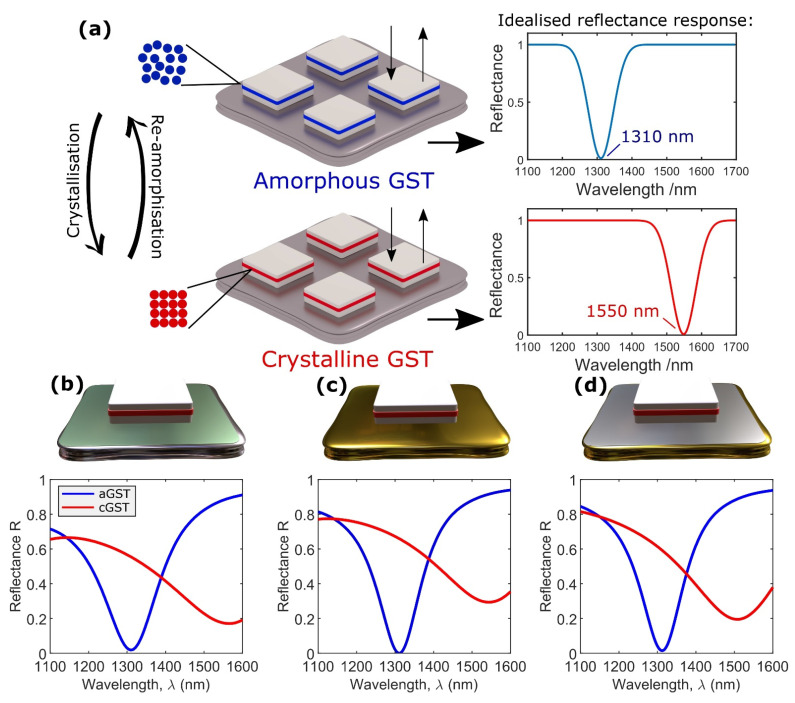
(**a**) Concept of device operation showing idealized reflectance spectra in amorphous and crystalline states. (**b**–**d**) Simulated amorphous and crystalline reflectance spectra for optimized device dimensions: (**b**) Al/Al_2_O_3_ bottom plane (Design 1), (**c**) Au bottom plane (Design 2), and (**d**) Au/Si_3_N_4_ bottom plane (Design 3).

The COMSOL model was used to optimize the dimensions and periodicity (pitch) of the unit cells, and the thicknesses of the Si and GST layers. For each of the three designs shown in Figure 3a, we obtained resonant absorption in telecommunication bands O and C (specifically, at λ_1_ = 1310 nm and at λ_2_ = 1550 nm) for the GST layer in the amorphous and crystalline states, respectively. Optimization was also carried out to maximize reflectance modulation depth ∆R upon switching the GST between the amorphous and crystalline states (where ∆*R = │R_a-GST_* − *R_c-GST_│*), as generically depicted in Figure 3a. Additional details about the optimization routine, design constraints, and analysis of the metasurface resonant behavior are provided in the supplementary *Section S1* (*Figures S1 and S2*) and in [25]. 

Figure 3b–d show the simulated optical performance (reflectance) of the three optimized designs for the amorphous and crystalline states of the GST layer. The designs behaved much as expected, with near-perfect absorption in the O band (1310 nm) for amorphous GST, and strong absorption in the C band (1550 nm) for crystallized GST. Perfect absorption in the C band was not physically possible, as the critical coupling condition could not be simultaneously satisfied for the two GST phases under the same device geometry [17,38]. Table 1 shows the final device dimensions for the three types of considered metasurfaces, and the modulation depths in reflection at the two selected bands. 

### 2.3. Hybrid-Metasurface Fabrication

Arrays of hybrid phase-change nanocubes were fabricated in areas of 120 µm × 120 µm on SiO_2_/Si substrates (previously cleaned with acetone and isopropyl alcohol) as follows (we start by describing the fabrication of Design 3, as it is the most complex):

1. For Design 3, an Au/Si_3_N_4_/Si/GST/Si layer stack was sputtered onto a clean substrate. A thin film of titanium (~20 nm) was included between substrate and gold layer in order to improve gold adhesion to the substrate and avoid its delamination. Direct-current (DC) sputtering (40 W) in an Ar atmosphere (10 sccm) was used for the metallic layers (i.e., Au and Ti). Sputtering pressure and base vacuum were 1.0 × 10^−3^ and 1.0 × 10^−6^ mbar, respectively. The Si_3_N_4_ layer was then deposited via radio-frequency (RF) sputtering (25 W) from a silicon nitride target, again in an Ar atmosphere (10 sccm, base pressure 1.0 × 10^−6^ mbar, sputtering pressure 1.5 × 10^−3^ mbar). Lastly, the remaining layers (i.e., silicon and GST) were deposited onto the Si_3_N_4_-coated gold plane. RF (200 W) sputtering was used for the silicon layers, and DC (25 W) sputtering for the GST. Sputtering pressure and base vacuum were the same as for the gold and titanium layers. The fabrication of Design 2 followed the same process as that for Design 3 but without incorporating the Si_3_N_4_ barrier layer. Design 1 was fabricated in the same way as Design 2 was, but replacing Au with Al.
2. After the relevant layers had been deposited, samples for all designs were covered with a polymer adhesion layer (Ti-Prime) employing a spin-coating machine at 4000 rpm for 20 s, with subsequent postbaking at 90 °C for 5 min. A negative resist (ma-N 2403) was then spin-coated at 3000 rpm for 60 s and postbaked at 90 °C for 10 min.
3. The patterns for the desired unit cell arrays were then transferred to the resist via e-beam lithography (Nanobeam NB4), with subsequent development in MF-319 solution for 45 s to eliminate the unexposed areas. After lithography, samples were postbaked at 90 °C for 5 min to increase the hardness of the remaining exposed areas.
4. Samples were then treated with a reactive-ion etching (RIE) process in a CHF_3_/SF_6_/O_2_ plasma mixture to remove regions not covered by the resist. Etching parameters were the same as those employed in [9]. Devices were then soft-sonicated in acetone to remove any excess resist.

**Figure 4:**
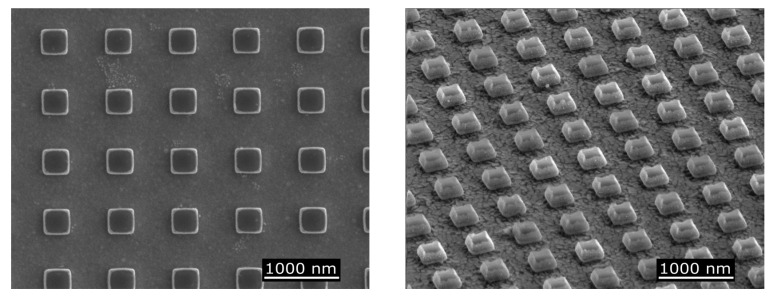
SEM images of typical as-fabricated cubic Si–GST–Si resonator structures (here with Au ground plane), taken (**left**) at normal incidence and (**right**) tilted at a 52° angle.

Scanning electron microscopy (SEM) was used to verify the consistency, morphology, and dimensions of the fabricated structures. Figure 4 shows SEM images of the Design 2 (Au bottom plane with no barrier layer) devices. Resonator widths were measured as 300 nm (±20 nm) and the pitch of the devices as 710 nm (±20 nm), both of which agreed with the desired geometry (as in Table 1). 

## 3. Results

**Figure 5:**
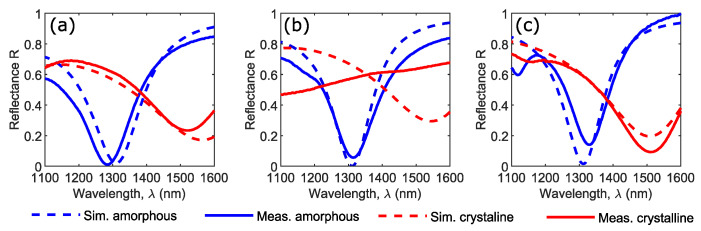
Measured (solid lines) and simulated (dashed lines) reflectance spectra of Si–GST–Si hybrid metasurface devices with GST layer in amorphous (blue lines) and crystalline (red lines) phases for (**a**) Al/Al_2_O_3_ bottom plane (Design 1), (**b**) Au bottom plane (Design 2), and (**c**) Au/Si_3_N_4_ bottom plane (Design 3).

To assess the performance of the as-fabricated hybrid metasurface devices, reflectance measurements were performed using a microspectrophotometer (JASCO MSV-5300, JASCO Corporation, Tokyo, Japan) between 1100 and 1600 nm over a spot diameter of 50 µm with an objective lens of NA 0.2 (i.e., excitation and collection angles from −12° to 12°). Devices were first measured in their pristine (as-deposited) amorphous phase, and then again after the GST layer had been crystallized by annealing it on a hot plate at 200 °C for 10 min (“static” crystallization to the cubic phase of GST occurs typically between 160 to 180 °C, depending on thin-film density and sputtering conditions [39]). Measured reflectance spectra of the three designs for both amorphous and crystalline states of the GST layer were compared to the simulations and are shown in Figure 5.

For Design 1 (Al/Al_2_O_3_ bottom plane, Figure 5a), the minimal reflectance achieved when the GST was amorphous (i.e., in the O band) showed almost perfect absorption (in line with our simulations), with only 2% reflectance at λ_1_ ~ 1310 nm. After the GST layer had been crystallized, a minimal reflectance of 23% was achieved in the C band (λ_2_ ~1550 nm), in agreement with our numerical modelling results. There were no signs of degradation of the optical response after the device had been annealed (crystallized). 

For Design 2 (Au bottom plane without barrier layer, Figure 5b), the as-fabricated (i.e., amorphous phase GST) devices were consistent with the simulated reflectance data, giving a minimum of 6% reflectance at 1315 nm (only 5 nm from the target wavelength of 1310 nm for minimal reflectance). However, after being annealed at 200 °C for 10 min, the expected resonant behavior entirely disappeared, which resulted in a near-flat spectrum with reflectance decreased towards shorter wavelengths. The disappearance of resonance here was most likely due to thermally activated diffusion of Au into the Si/GST/Si resonator stack (as pointed out in Section 1, Au readily diffuses into both Si [21,22] and GST [20]).

Lastly, results for Design 3 (where we incorporated an 8 nm thick Si_3_N_4_ thermal diffusion layer between Au bottom plane and Si/GST/Si resonators) are shown in Figure 5c. Here, again, the amorphous phase showed good consistency with the simulations; the fabricated devices had an absorption minimum of 14% reflection in the O band (λ_1_ ~1310 nm). Contrary to Design 2, the expected optical performance was maintained upon crystallization with a 9% reflectance minimum in the C band (λ_1_ ~ 1510 nm). This highlighted the importance of thermal-diffusion barrier layers in thermally tunable Si or hybrid Si/GST metasurfaces when using Au as the plasmonic metal. Specifically for Design 3 (Figure 5c), a secondary small experimental absorption peak appeared at shorter wavelengths. This was related to the fact that the reflectance spectra were not experimentally measured with normally incident light (as used for the simulations), but using light spread over a range of approximately ±12 degrees due to the focusing effect of the 0.2 NA objective lens of the microspectrophotometer. Additional details on the angular behavior of the reflectance spectra are in supplementary information (*Section S2, device performance at oblique incidence,* *Figure S3*). 

## 4. Discussion and Conclusions

Due to its excellent plasmonic properties and relatively high melting point compared to those of other common plasmonic materials, Au was the metal of choice for the development of tunable phase-change-based plasmonic and hybrid dielectric–plasmonic metasurfaces. In such approaches, the Au layer performed the dual role of providing an in situ electrical heater for switching the phase-change layer. However, we showed that the thermally induced diffusion of Au into dielectric meta-atoms has a catastrophic, irreversible, and deleterious impact on the optical response of the metasurface. Fortunately, such unwanted effects can be overcome by the inclusion of an appropriate barrier layer (here specifically Si_3_N_4_) in the metasurface design. We elucidated this by carrying out a systematic study consisting of the design, fabrication, and characterization of three different dual-band absorbers/switches based on hybrid silicon/PCM metasurfaces lying on bottom metal planes that could simultaneously act as low-loss reflective elements and electrical resistive heaters. Each design had a different bottom-plane configuration (namely, Al/Al_2_O_3_, Au, and Au/SiN) in order to clarify and investigate the effects of diffusion under different scenarios. As proof of concept, the three structures were numerically optimized to be switched between strong absorption resonances at telecommunication wavelengths of 1310 and 1550 nm upon switching the GST layer between its amorphous and crystalline states. Modulation performance of the as-fabricated devices was very good, with extinction ratios ranging from −5.0 to −9.3 dB, and insertion losses from 0.2 to 2.2 dB, which favorably compares to values reported in the literature [40] (the definition of extinction ratio and insertion loss for our devices is defined in the supplementary information). Results from the experimental metasurfaces with an Al/Al_2_O_3_ bottom plane showed excellent consistency with theoretical calculations and no signs of degradation of the optical response upon GST crystallization. However, the melting point of Al is only marginally above that of commonly used PCM alloys, so Al is not well-suited for use as an in situ electrical heater for switching (specifically for amorphizing) GST layers embedded in metasurfaces. Our results with Au bottom planes showed good consistency with simulated and measured optical responses for the amorphous phase of the GST layer, but suffered from dramatic degradation of the expected optical performance upon GST crystallization as a consequence of the thermally activated diffusion of gold into the Si/GST/Si resonators. However, the incorporation of an ultrathin Si_3_N_4_ barrier layer between Au bottom plane and Si/GST/Si resonators prevented any such degradation, and retained expected optical performance upon crystallization of the GST layer.

In summary, we successfully demonstrated the use of barrier layers in thermally tunable metasurfaces to prevent the thermally activated diffusion of plasmonic metals at temperatures required for GST crystallization. In this context, we also showed how diffusion into Si/GST should not be ignored in phase-change metasurface design and development due to its dramatic and irreversible negative impact on the metasurface’s optical response. 

## Figures and Tables

**Table 1:** Optimized device dimensions and modulation depth of three considered designs.

Parameter	Design 1	Design 2	Design 3
Pitch	719 nm	705 nm	758 nm
Cube width	386 nm	293 nm	300 nm
Height Si (bottom)	33 nm	34 nm	37 nm
Height GST	36 nm	37 nm	40 nm
Height Si (top)	27 nm	29 nm	31 nm
Oxide/Si_3_N_4_ thickness	∼4 nm	N/A	8 nm
∆R, λ = 1310 nm (O band)	56%	66%	60%
∆R, λ = 1550 nm (C band)	72%	62%	67%

## Data Availability

Data supporting results discussed in this manuscript is openly available in the University of Exeter’s repository, ORE.

## Supplementary Materials

The following are available online at https://www.mdpi.com/2079-4991/11/2/525/s1, Figure S1: Reflectance of devices at 1310 nm for varying resonator stack widths and pitch with GST in its amorphous phase, Figure S2: Electric and magnetic field distribution of Al devices in resonance, Figure S3: Angular dependence of the reflectance spectra for Designs 1 and 3.
