# Supplementary material for: Enhanced Performance and Diffusion Robustness of Phase-Change Metasurfaces via a Hybrid Dielectric/Plasmonic Approach
Source: Nanomaterials (Basel). 2021 Feb 18;11(2):525. doi: 10.3390/nano11020525 (PMC7922528; doi:10.3390/nano11020525)
Supplement: Supplementary file 1 [file nanomaterials-11-00525-s001.zip › nanomaterials-1111898-supplementary.pdf]

nanomaterials

Supplementary Material

Enhanced Performance and Diffusion Robustness of Phase-Change Metasurfaces via a Hybrid Dielectric/Plasmonic Approach

Joe Shields [1,†], Carlota Ruiz de Galarreta [1,†], Jacopo Bertolotti [1] and C. David Wright [1,*]

[1] University of Exeter; College of Engineering Mathematics and Physical Sciences, Exeter EX4 4QF, UK; js1116@exeter.ac.uk (J.S.); cr408@exeter.ac.uk (C.R.d.G.); j.bertolotti@exeter.ac.uk (J.B.); david.wright@exeter.ac.uk (C.D.W.)

* Correspondence: david.wright@exeter.ac.uk

† These authors contributed equally to this work.

Citation: Shields, J.; de Galarreta, C.R; Bertolotti, J.; Wright, C.D. Enhanced Performance and Diffusion Robustness of Phase-Change Metasurfaces via a Hybrid Dielectric/Plasmonic Approach. *Nanomaterials* **2021**, *11*, 525. https://doi.org/10.3390/nano11020525

Academic Editor: Andrey B. Evlyukhin

Received: 30 January 2021

Accepted: 15 February 2021

Published: 18 February 2021

Publisher's Note: MDPI stays neutral with regard to jurisdictional claims in published maps and institutional affiliations.

Copyright: © 2021 by the authors. Licensee MDPI, Basel, Switzerland. This article is an open access article distributed under the terms and conditions of the Creative Commons Attribution (CC BY) license (http://creativecommons.org/licenses/by/4.0/).

S1. Metasurface Optimization and Analysis

(a) Device Optimization

Optimizations and parameter sweeps for the three devices were performed in Comsol Multiphysics ® to obtain the required device geometry. Figures S1a,c,e show (for Al, Au and Au with Si_3N_4 devices respectively) the reflectance at 1310 nm with GST in its amorphous phase as the pitch and stack widths were varied. Stacks were set to 100 nm in height with equal layer thickness of Si, GST and Si, as thicker cubes were found to decrease the amount of absorption significantly, which is not suitable for the kind of devices proposed. This was interpreted as a decoupling of the electric field enhancement inside the cubic resonators with the bottom metal plane, resulting in minimization of plasmonically induced losses (i.e. resonances changing from plasmonic to purely dielectric). From Figures S1a,c,e, minimum regions were found in reflectance at a pitch of between 700 nm and 1000 nm and a stack width of approximately 400 nm in the case of Al and 300 nm in the cases of Au and Au and Si_3N_4.

The thickness of the crystalline GST was then varied to explore the amount of material required for switching between the two considered bands upon crystallization (i.e. 1310 nm and 1550 nm). As can be seen in the parametric sweep in Figure S1b,d,f for Al, Au, and Au with Si_3N_4 devices respectively, increasing the volume of crystalline GST in the stack will shift the resonance to higher frequencies and will also broaden the resonance (lower Q-factor) due to the characteristic increase of the optical losses of GST upon crystallization. It was found that a GST thickness of approximately 35 nm shifts the absorption resonance into the C-band for Al devices and 40 nm is required for Au and Au with Si_3N_4.

With these basic parameters as a starting point, small changes were performed to increase the absorption and modulation depths at the desired wavelengths. Final device dimensions are shown in table 1 from the main text.

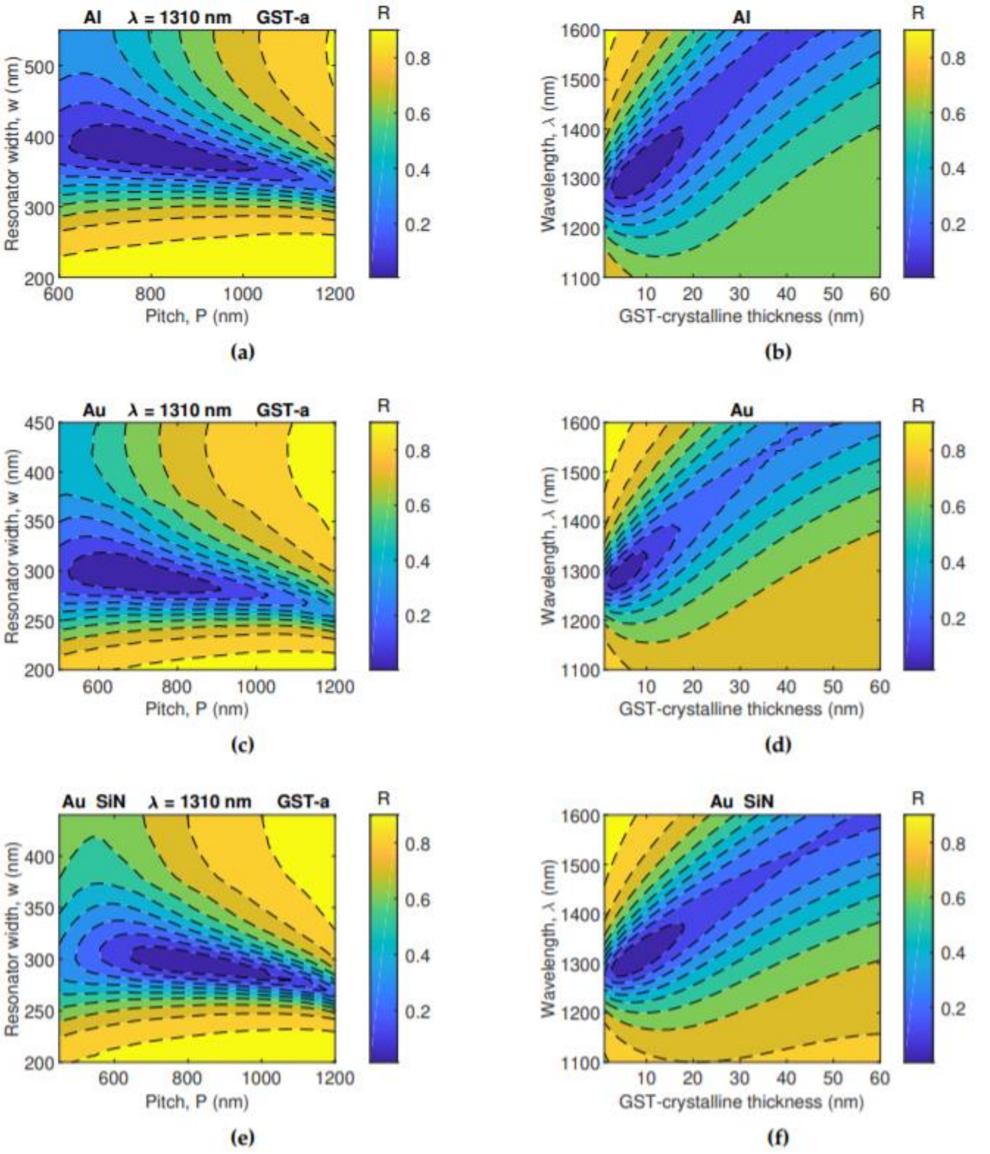

Figure S1. Reflectance of devices at 1310 nm for varying resonator stack widths and pitch with GST in its amorphous phase with an Al (**a**), Au (**c**) and Au with Si_3N_4 (**e**) ground plane. Stack height set to 100 nm with equal thickness of Si, GST and Si. Reflectance spectrum varying the thickness of the crystalline GST layer in the resonators for Al (**b**), Au (**d**), and Au with Si_3N_4 (**f**) ground plane.

(b) Device Resonant Behavior

Figure S2 shows typical simulation results (in this case for Al devices) of the normalized magnitude of the out of plane magnetic field component $|H_y|$, and the electric field's direction and relative magnitude, represented by arrows. The field distributions show the excitation of a transverse-magnetic dipole-like plasmonic resonance for both amorphous and crystalline phases, driven by displacement and conduction current loops at the dielectric and metal interfaces respectively. As can be seen, this mode in both the amorphous and crystalline phases is similar in character but occurs at different spectral position, namely 1310 nm in the amorphous phase and 1550 nm in the crystalline phase.

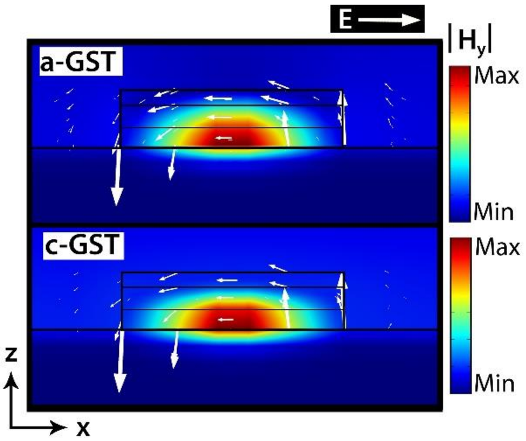

Figure S2. Simulation of Al devices' normalized magnitude of the magnetic field component $|H_y|$ showing magnetic dipole mode at resonance at 1310 nm and 1550 nm for amorphous and crystalline phases respectively. Arrows indicate the electric field direction and relative magnitude.

(c) Device Extinction Ration (ER) and Insertion Loss (IL)

The devices developed in the main manuscript can operate as modulators at wavelengths in both the O- and C- telecommunication bands. Common performance metrics for modulators are extinction ratio (ER) and insertion loss (IL). ER and IL for our devices are defined according to standard practice as follows (and quantitative values for as-fabricated devices are given in the main text):

$$ER = -10\log_{10}(R_{cry}/R_{am}), \text{ in the O-band}$$

$$ER = -10\log_{10}(R_{am}/R_{cry}), \text{ in the C-band}$$

$$IL = -10\log_{10}(R_{cry}), \text{ in the O-band}$$

$$IL = -10\log_{10}(R_{am}), \text{ in the C-band}$$

where Rcry and Ram refer to the reflectance of the device when the PCM layer is the crystalline and amorphous states respectively

S2. Device Performance at Oblique Incidence

In order to understand the secondary weaker absorption peaks observed experimentally at shorter wavelengths for Au and Au/Si_3N_4 based devices, the reflectance spectrum at oblique incidence was numerically evaluated within the angular range defined by the numerical aperture of the spectrometer microscope (i.e. excitation and collection angles from −12° to 12°, NA 0.2). For comparison, the angular response of Al/Al_2O_3 devices was also numerically computed, both of them for the amorphous phase of GST. In Figure S3a we show the reflectance spectrum as a function of the angle of incidence (AOI) for the case of Al/Al_2O_3 devices. As it can be seen from the arrows, an additional low Q resonant mode falling at λ ~ 1150 nm can be excited via off-normal incidence. This results, as shown in Figure S3b, in a slightly different reflectance spectrum at shorter wavelengths when averaging the contribution of all angles of incidence (blue solid curve) to the optical response, which in the case of Al/Al_2O_3 devices is fairly similar to the optical response at pure normal incidence (i.e. AOI = 0°, black dashed curve). This is due to both the low Q nature of the shorter wavelength mode, as well as its proximity to the main absorption band located at λ = 1310 nm, which results in smoothing/overlapping of both modes when averaging the optical response.

In Figure S3c, we now show the reflectance spectrum as a function of the AOI for the case of Au/Si_3N_4 devices. Similar to Al-based devices, an additional mode can be excited off-axis, but here at even shorter wavelengths around λ ~ 1090 nm. However, due to lower plasmonic losses present in Au (cf. Aluminum), the Q factors of the main resonant (λ =

1310nm) and off-axis (λ = 1090 nm) modes are here clearly higher, which results in both modes being well spectrally separated. As shown in S3d, the off-axis mode can be therefore appreciated when computing the average optical response (blue curve), causing major differences with respect to the optical response at normal incidence (black dashed curve) when compared to Al devices.

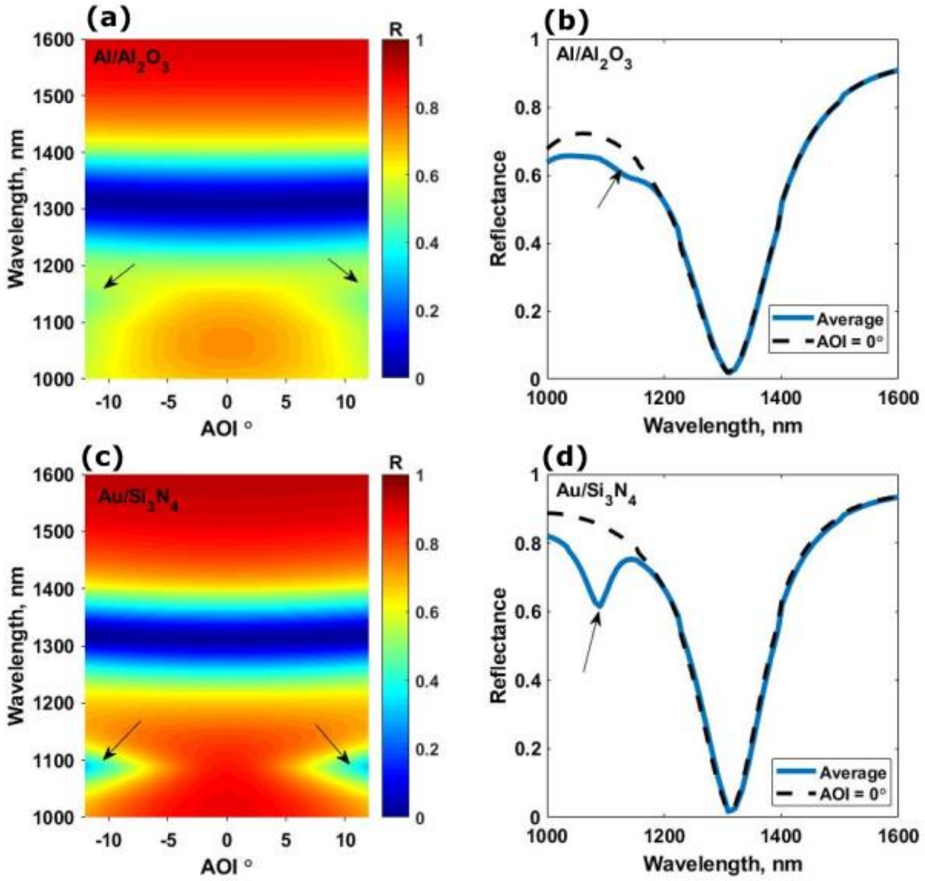

Figure S3. (**a**) Reflectance spectrum (colorbar) as a function of the angle of incidence for amorphous Al/Al_2O_3 devices. (**b**) Reflectance spectrum of amorphous Al/Al_2O_3 devices for: AOI = 0° (dashed black curve) and averaged across the angles defined by the numerical aperture of the microspectrometer lens (blue curve). (**c**) Reflectance spectrum (colorbar) as a function of the angle of incidence for amorphous Au/Si_3N_4 devices. (**d**) Reflectance spectrum of amorphous Au/Si_3N_4 devices for: AOI = 0° (dashed black curve) and averaged across the angles defined by the numerical aperture of the microspectrometer lens (blue curve).
